# Highly conductive nano-sized Magnéli phases titanium oxide (TiO_x_)

**DOI:** 10.1038/s41598-017-03509-y

**Published:** 2017-06-16

**Authors:** Aditya F. Arif, Ratna Balgis, Takashi Ogi, Ferry Iskandar, Akihiro Kinoshita, Keitaro Nakamura, Kikuo Okuyama

**Affiliations:** 1 Department of Chemical Engineering, Graduate School of Engineering, Hiroshima University, 1-4-1 Kagamiyama, Higashi-Hiroshima, 739-8527 Japan; 2 Department of Physics, Institut Teknologi Bandung, Jalan Ganesha No. 10 Bandung, 40132 West Java, Indonesia; 3 Research Center for Production and Technology, Nisshin Seifun Group Inc., 5-3-1, Tsurugaoka, Fujimino City, Saitama 356-8511 Japan

## Abstract

Despite the strong recent revival of Magnéli phase TiO_x_ as a promising conductive material, synthesis of Magnéli phase TiO_x_ nanoparticles has been a challenge because of the heavy sintering nature of TiO_2_ at elevated temperatures. We have successfully synthesized chain-structured Magnéli phases TiO_x_ with diameters under 30 nm using a thermal-induced plasma process. The synthesized nanoparticles consisted of a mixture of several Magnéli phases. A post-synthesis heat-treatment was performed to reduce the electrical resistivity without changing the particle morphology. The resistivity of the heat-treated particle was as low as 0.04 Ω.cm, with a specific surface area of 52.9 m^2^ g^−1^. The effects of heat-treatment on changes in the crystal structure and their correlation with the electron conductivity are discussed based on transmission electron microscopy images, X-ray diffraction spectra, and X-ray adsorption fine structure spectra. Electrochemical characterization using cyclic voltammetry and potentiodynamic scan shows a remarkable electrochemical stability in a strongly oxidizing environment.

## Introduction

Substoichiometric titanium oxides, chemical formula Ti_*n*_O_2*n*−1_ (*n* = 4–9), are often referred to as Magnéli phase TiO_*x*_ and have attracted much recent attention because of growing demand for conductive materials. Magnéli phase TiO_x_ was studied for the first time in the 1950s by the group of Arne Magnéli following the construction of a phase diagram of a titanium-oxygen system by De Vries *et al*.^1^. The electrical properties of these materials were then studied by Bartholomew *et al*., revealing a semiconductor-to-metal transition at certain temperatures and a decrease in conductivity with an increase in oxygen content^2^. Because of the oxygen deficiency, which results in delocalized electrons in the *d* band, some crystallographic shear structures are regularly introduced in the rutile structure and act as a good electron pathway. Therefore, Magnéli phase TiO_x_ has a high electrical conductivity that is comparable to carbon^3^. However, its advantage over carbon is that it is known to be durable in electrochemically oxidizing environments^4, 5^.

In recent years, several efforts have been devoted to synthesizing Magnéli phase TiO_x_, typically by the reduction of rutile titania (TiO_2_) under high temperatures between 600 and 1000 °C. According to the titanium-oxygen system phase diagram, this is the simplest pathway to obtain Magnéli phase TiO_x_. Commonly employed reducing agents include carbon^6, 7^, zirconium^8^, and hydrogen (H_2_) or ammonia^9, 10^. These reduction methods have been proven to effectively synthesize single Magnéli phase TiO_x_, often Ti_4_O_7_ which has the highest conductivity among all Magnéli phases^11^. However, the high reduction temperature promotes sintering, which usually starts at approximately 700 °C or lower for TiO_2_ nanoparticles (NPs)^12^. Because of heavy sintering, the diameter of the resulting TiO_x_ particles commonly range from 500 nm to 1 μm, with surface areas of approximately 25 m^2^ g^−1^ or lower. Considering that a high specific surface area is important in many applications, improvement in the surface area of the synthesized Magnéli phase TiO_x_ becomes a challenge. One popular approach to increase the surface area is by reducing the particle sizes to the nano scale. Nano sizes also enable the particles to be effectively packed, hence reducing the contact resistance between particles. In light of this, Ioroi and his group made several attempts to reduce the TiO_x_ particles size, e.g., by employing pulsed UV laser irradiation or mechanical grinding^5, 13^. However, the surface areas of the resulting particles were still below 20 m^2^ g^−1^, although the particle diameter was reduced to approximately 70 nm. Another synthesis approach carried out by Portehault *et al*. was a sol-gel method to synthesize monoliths consisting of TiO_x_ clusters in a carbon matrix, resulting in a surface area as high as 400 m^2^ g^−1^ ^14^. Although the carbon matrix contributed to a high surface area, the proportion of carbon was very high whereas TiO_x_ NPs, with their absence or minimum presence of a carbon matrix, are favorable for improved durability.

The present work reports a successful synthesis of chain-structured Magnéli phases TiO_x_ NPs using an induction thermal plasma method. To date, this is the first report on the synthesis of Magnéli phases TiO_x_ NPs under 30 nm in diameter. The same synthesis methods have previously been used for the synthesis of α′′-Fe_16_N_2_/Al_2_O_3_ core-shell^15^ and various metal oxide NPs^16^. Conductivity of the synthesized-TiO_x_ NPs’ is analyzed based on the crystal structure. Their electrochemical stability in oxidizing environment is studied to assess the feasibility of the plasma-synthesized TiO_x_ NPs application as conductive materials in many devices.

## Methods

**Figure 1:**
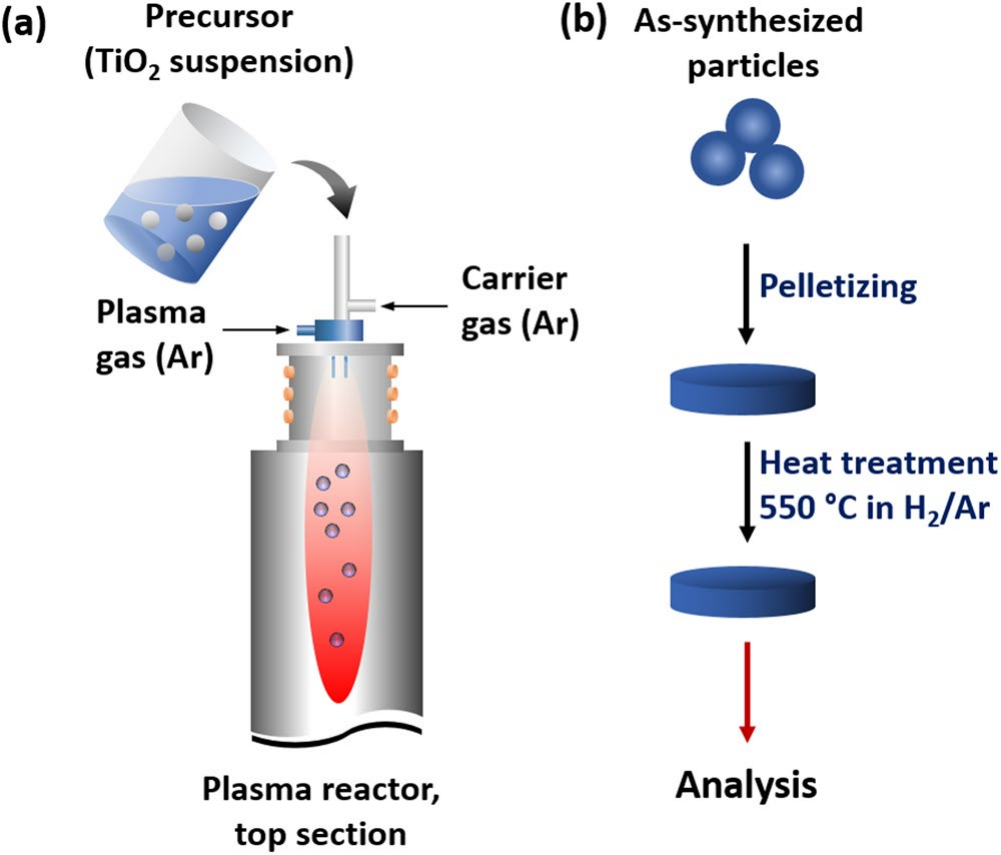
Illustration of (**a**) TiO_x_ NP preparation using RF induction thermal plasma method, and (**b**) post-synthesis heat treatment to improve the electrical conductivity.

Three samples of Magnéli phases TiO_x_ were synthesized using an RF induction thermal plasma method that has been reported in detail elsewhere^16^. Typically, a precursor suspension was made by dispersing rutile TiO_2_ particles with an average diameter (*d*_p,avg_) of 2 μm (Nisshin Engineering Inc., Tokyo, Japan) into a water-isopropyl alcohol (IPA) mixture. The mass ratio between TiO_2_, IPA, and water was 43:43:14, 42:42:16, and 39:39:22 for TiO_x_-A, TiO_x_-B, and TiO_x_-C, respectively. Figure 1 shows a schematic illustration of the experiment. The suspension was then fed into a plasma reactor with argon (Ar) used as both the plasma and carrier gas [Fig. 1(a)]. To improve the conductivity, a set of thermal conditioning experiments was performed to the as-synthesized samples [Fig. 1(b)]. The sample NPs were first pelletized by applying 50 MPa pressure to 0.5 g of the powder in a pelletizing template. The pellet, having dimensions of 15 mm in diameter and 1 mm in thickness, was then heated under 3% H_2_ with an argon balance in a vacuum furnace (μBF, Koyo Thermo Systems Co., Ltd., Nara, Japan) for 1 hour.

The morphological structure of the particles was observed using a field-emission scanning electron microscope (SEM; S-5000, 20 kV, Hitachi High-Tech. Corp., Tokyo, Japan) and transmission electron microscope (TEM; JEM-2010, 200 kV, JEOL Ltd., Tokyo, Japan). The crystal structures of all samples were evaluated using X-ray diffraction (XRD; Bruker D2 Phaser, Bruker AXS GmbH, Karlsruhe, Germany), while the oxidation state of the particles was examined using X-ray Adsorption Fine Structure (XAFS; BL5S1, Aichi Synchrotron Radiation Center, Seto, Japan). Anatase TiO_2_ and Ti_2_O_3_ (Sigma Aldrich, St. Louis, MO, USA) were used as the reference for XAFS analysis. Semi-quantitative analysis on the chemical structure composition was performed using Diffrac. Eva 3.0 software (Bruker AXS GmbH, Karlsruhe, Germany) based on the XRD spectral intensities. Based on the N_2_ adsorption ability (BELSORP-max, MicrotracBEL Corp., Osaka, Japan), the surface areas of the particles were calculated by the Brunauer-Emmett-Teller (BET) method. The surfaces of the particles were evaluated using Fourier-transform infrared (FT-IR) spectroscopy (Spectrum One, Perkin Elmer Inc., Waltham, MA, USA). The electrical resistivity of the sample before and after heat-treatment was measured using the four-probe method (Loresta-GP, Mitsubishi Chemical Analytech Co., Ltd., Kanagawa, Japan). For better accuracy, the electrical resistivity of each sample was measured three times to three different pellets. The measurement was conducted within a short period in a controlled ambient. Therefore, we may suppress the effect of pellet inhomogeneity and the difference in the atmospheric conditions.

TiO_x_ NPs stability in an oxidizing environment was evaluated by electrochemical analytical methods using a potentiostat (HR-301, Hokuto Denko Corp., Tokyo, Japan) in a 3-electrode configuration. Platinum wire and a reversible hydrogen electrode (RHE) was used as the counter and reference electrode, respectively. Cyclic voltammetry and potentiodynamic scan were conducted in 1 M HCl solution (Cica-reagent, Kanto Chemical Co. Inc., Tokyo, Japan) saturated with oxygen with a potential sweep of 50 mV s^−1^. The potential window for cyclic voltammetry and potentiodynamic scan was 0 to 1.4 V/RHE and 0 to 1.5 V/RHE, respectively. 1.4 V/RHE is the maximum theoretical potential of a fuel cell during start and stop conditions. This potential was chosen as the cyclic voltammetry reverse potential to represent a severe environment that might be imposed to TiO_x_ NPs in their application as conductive material. The active material ink was prepared by dispersing TiO_x_ NPs (2.64 mg) in a mixture of ultrapure water (0.95 ml), 2-propanol (0.3 ml; Cica-reagent, Kanto Chemical Co. Inc., Tokyo, Japan), and Nafion® (5 μl; Wako Pure Chemical Industries Ltd., Osaka, Japan) in an ultrasonic bath. The corrosion rate (*r*) was calculated based on the exchange current density using a derivation of Faraday’s equation (Eq. 1). *i*, *a*, *n*, and F is exchange current density, average molecular weight of TiO_x_, number of electrons, and Faraday constant, respectively.

1 $$r=\frac{i{\rm{M}}}{n{\rm{F}}}$$

## Results and Discussion

**Figure 2:**
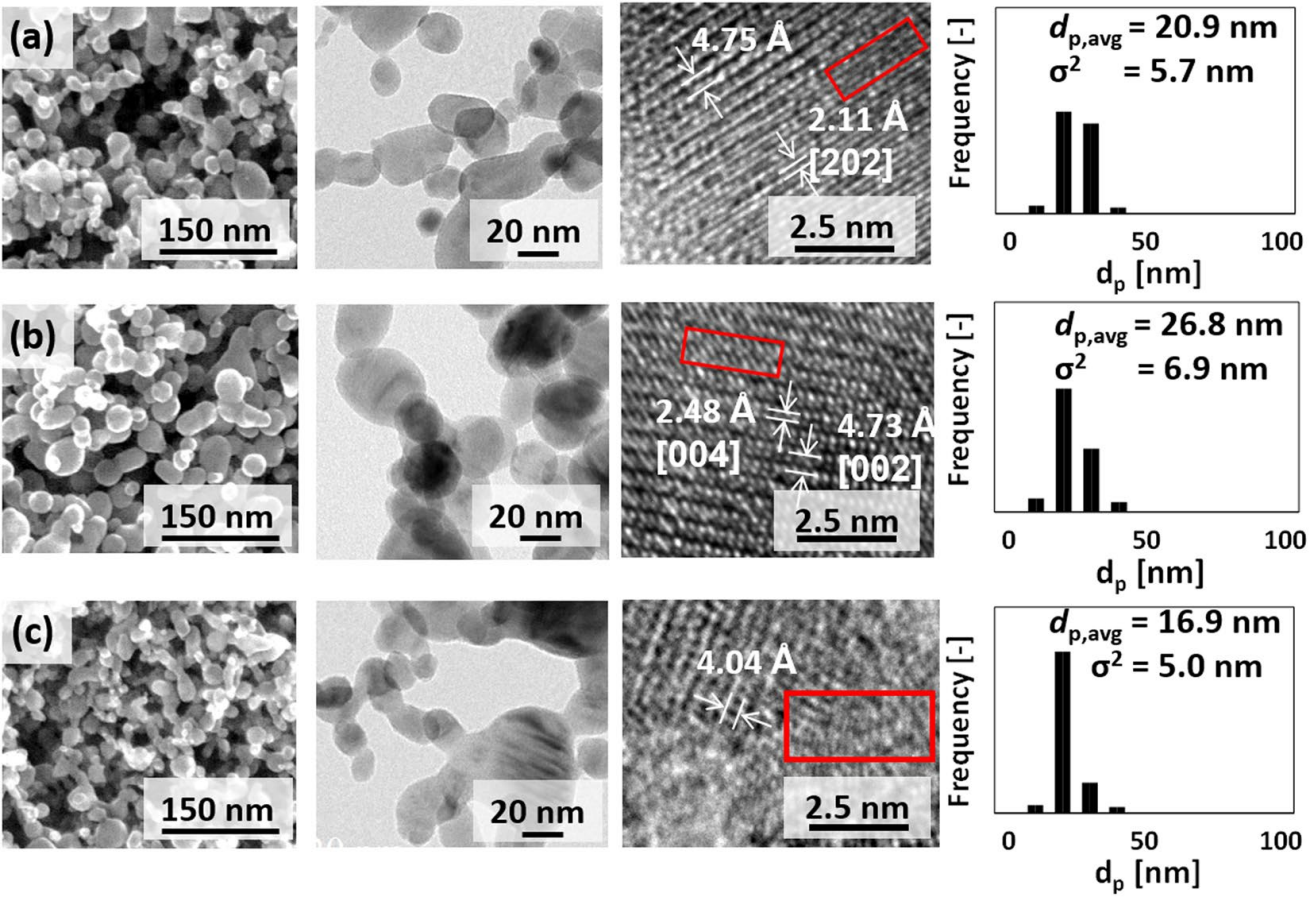
SEM and TEM images showing the chain structure and the lattice structure of the NPs, and particle size distribution of (**a**) TiO_x_-A, (**b**) TiO_x_-B, and (**c**) TiO_x_-C. Red boxes in the TEM images indicate discontinuous lattices. Some lattice spaces can be associated with reduced titanium oxides, such as Ti_3_O_5_ [002] (4.73 Å), Ti_2_O_3_ [202] (2.11 Å), Ti_4_O_7_ [004] (2.48 Å).

Electron microscopy images of the as-synthesized particles are shown in Fig. 2. According to these images, the particles in all three samples had chain-like structures with a high degree of sphericity, as may be expected from a gas-phase synthesis method^17^. Topological measurement on the NPs shows a *d*_p,avg_ of 20.9 nm, 26.8 nm, and 16.9 nm for TiO_x_-A, TiO_x_-B, and TiO_x_-C, respectively. The size distribution of the NPs was narrow, indicated by a standard deviation of 5.7 nm, 6.9 nm, and 5.0 nm for TiO_x_-A, TiO_x_-B, TiO_x_-C, respectively. Images of high-resolution (HR) TEM analysis show a non-uniform lattice structure in all samples. The distance between the lattices are inhomogeneous, even between one lattice pair and its neighboring pair. Although it is normal for one Magnéli structure to have different distances between the Ti atoms, dependent on the type of Ti atom pairing^18^, variations in the lattice distance of these samples were random. Some lattice distances are very wide, for example, 4.75 Å, which is not associated to a specific Magnéli phase, or 4.73 Å, which might refer to the [002] crystal of Ti_3_O_5_ (Fig. 2b). Some other lattice distances may more easily be associated with some Magnéli phases, for example, 2.11 Å, which is associated with the [202] crystal of Ti_2_O_3_ (Fig. 2a), and 2.48 Å, which possibly corresponds to the [004] crystal of Ti_4_O_7_ (Fig. 2b). A precise estimation on the crystal structure is, however, difficult to make due to the presence of some dislocations, as indicated by the red boxes in Fig. 2.

**Figure 3:**
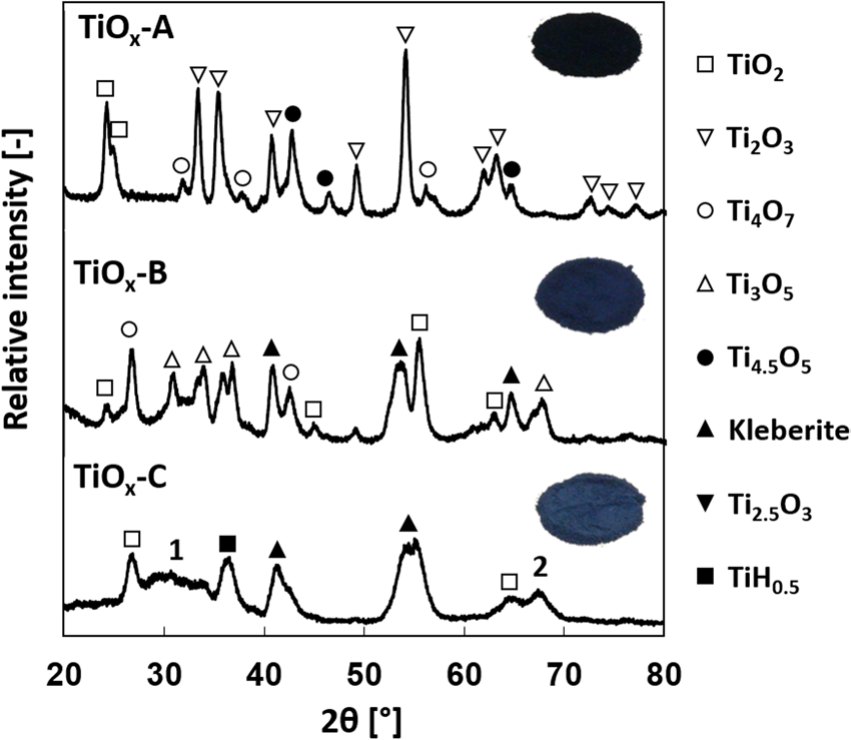
XRD spectra and the main peaks assignment of TiO_x_-A, TiO_x_-B, and TiO_x_-C. TiO_x_-A shows the greatest extent of reduction, indicated by the domination of the Ti_2_O_3_ phase. The broad peak indicated by **1** is assigned to Ti_3_O_5_, Ti_4_O_7_, Ti_8_O_15_, and TiO_2_ while **2** is assigned to Ti_3_O_5_ and TiO_2_. Samples with a greater extent of reduction tend to have darker colors. A complete list of the identified species is provided in the supporting information.

Confirming the results of the HR-TEM analysis, the XRD spectra of the as-synthesized samples (Fig. 3) show that TiO_x_-A, TiO_x_-B, and TiO_x_-C were mixtures of several Magnéli phases and the other titanium-based species. TiO_x_-A mainly consists of Ti_2_O_3_ and TiO_2_, while TiO_x_-B includes Ti_3_O_5_ and Ti_4_O_7_. In both TiO_x_-A and TiO_x_-B, some suboxides other than Magnéli phases were present. Unlike the spectra of the other two samples, where sharp peaks with high intensities are observed, the spectrum of TiO_x_-C shows broader peaks of lower intensity. This result agrees with the analysis of the HR-TEM images wherein TiO_x_-C contained some amorphous phases. TiO_2_, kleberite (Ti_0.374_O_0.5_(OH)_0.5_), and TiH_0.5_ are identified from the sharper peaks. The broad peak between 27° and 36° (**1**) is assigned to Ti_3_O_5_, Ti_4_O_7_, Ti_8_O_15_, and TiO_2_ while **2** is assigned to Ti_3_O_5_ and TiO_2_. Visually, the sample with a greater extent of reduction is indicated by particles that are darker in color.

The different extent of reduction likely corresponds to the content of IPA-derived carbon formed during the synthesis. Precursor of TiO_x_-A contained more IPA than that the other precursors did, resulting in the formation of more carbon during the synthesis. Since carbon can reduce TiO_2_, sample with the highest carbon content will have higher extent of reduction. This is also the reason for the formation of TiC in TiO_x_-A (Table S1 of supporting information).

**Figure 4:**
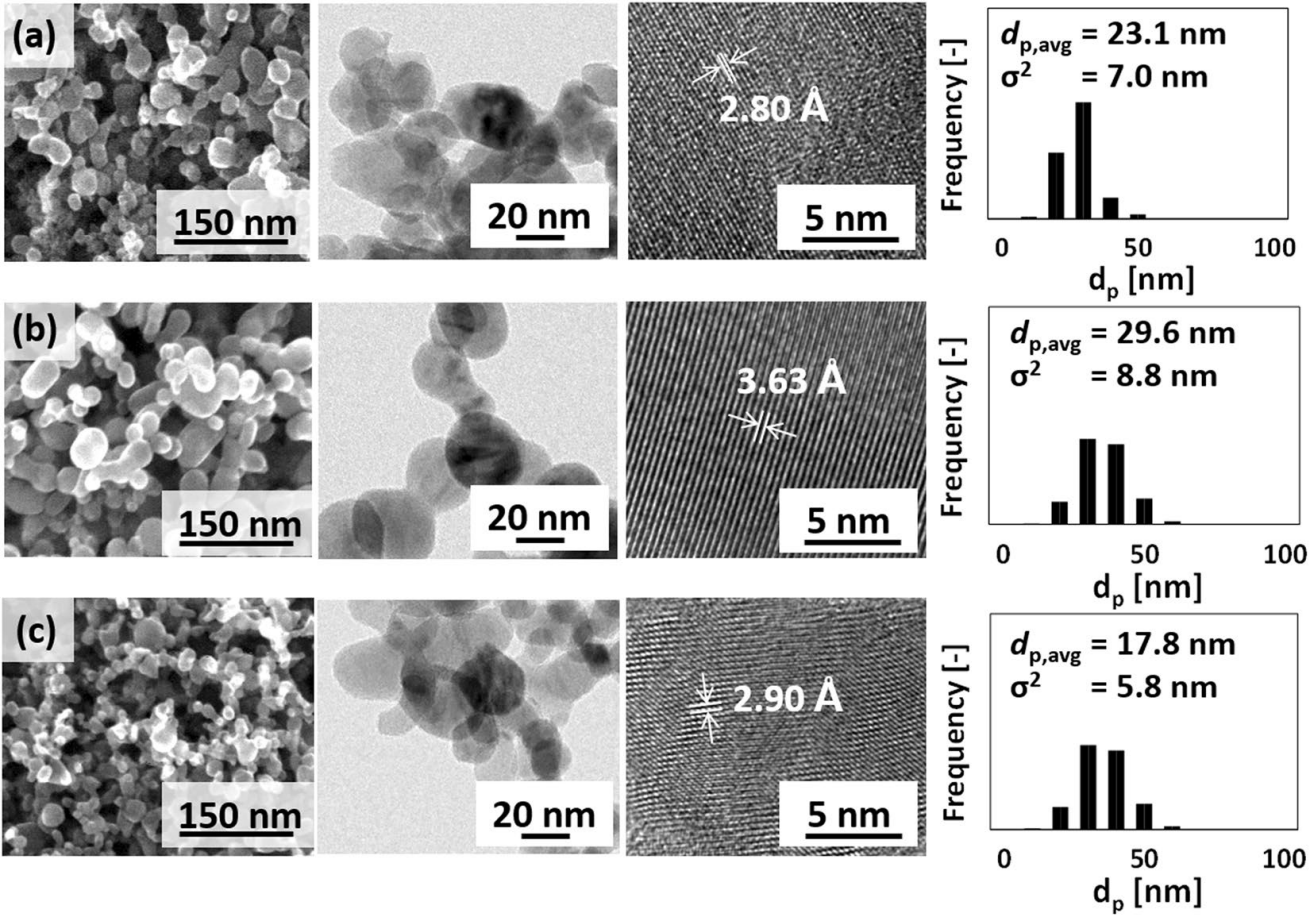
SEM, TEM images, and particle size distribution of heat-treated (**a**) TiO_x_-A, (**b**) TiO_x_-B, and (**c**) TiO_x_-C. A clear lattice structure can be observed for the heat-treated samples, showing Ti_4_O_7_ [022] (2.8 Å), Ti_4_O_7_ [122] (2.9 Å), and higher oxidation states TiO_x_ (likely Ti_8_O_15_ [10 $\overline{{\rm{11}}}$] for 3.63 Å).

Heat treatment conducted at 550 °C improved the quality of the lattice structure. Although the average particle diameters were slightly greater than those of the as-synthesized particles, Fig. 4 shows that they were within the margin of error. We can, therefore, say that the heat treatment did not significantly change the particle size or the morphology. The BET surface areas of heat-treated samples were 52.9, 44.2, and 55.2 m^2^ g^−1^ for TiO_x_-A, TiO_x_-B, and TiO_x_-C, respectively.

According to the HR-TEM images, the distances between the lattices of heat-treated NPs were uniform and the lattices were less discontinuous than the non-heat-treated particles. The clear lattice structure indicates increased crystallinity of the particles. Based on a randomized sampling of the HR-TEM images, some of the lattice distances were close to those associated for Ti_4_O_7_, especially for heat-treated TiO_x_-A (2.8 Å for Ti_4_O_7_ [022]) and TiO_x_-C (2.9 Å for Ti_4_O_7_ [112])^18^. The other identified lattice distances may be associated to Magnéli phases with higher oxygen content, such as Ti_8_O_15_ [10 $\overline{{\rm{11}}}$]. We may, therefore, expect the presence of a new Ti_4_O_7_ phase and the neighbouring structure in the heat-treated samples. It is interesting that the crystal structure changed at a temperature lower than that usually needed. The presence of residual energies in the as-synthesized NPs due to the involvement of the high synthesis energy in RF thermal induction plasma likely decreased the amount of external energy required to change the crystal structure.

**Figure 5:**
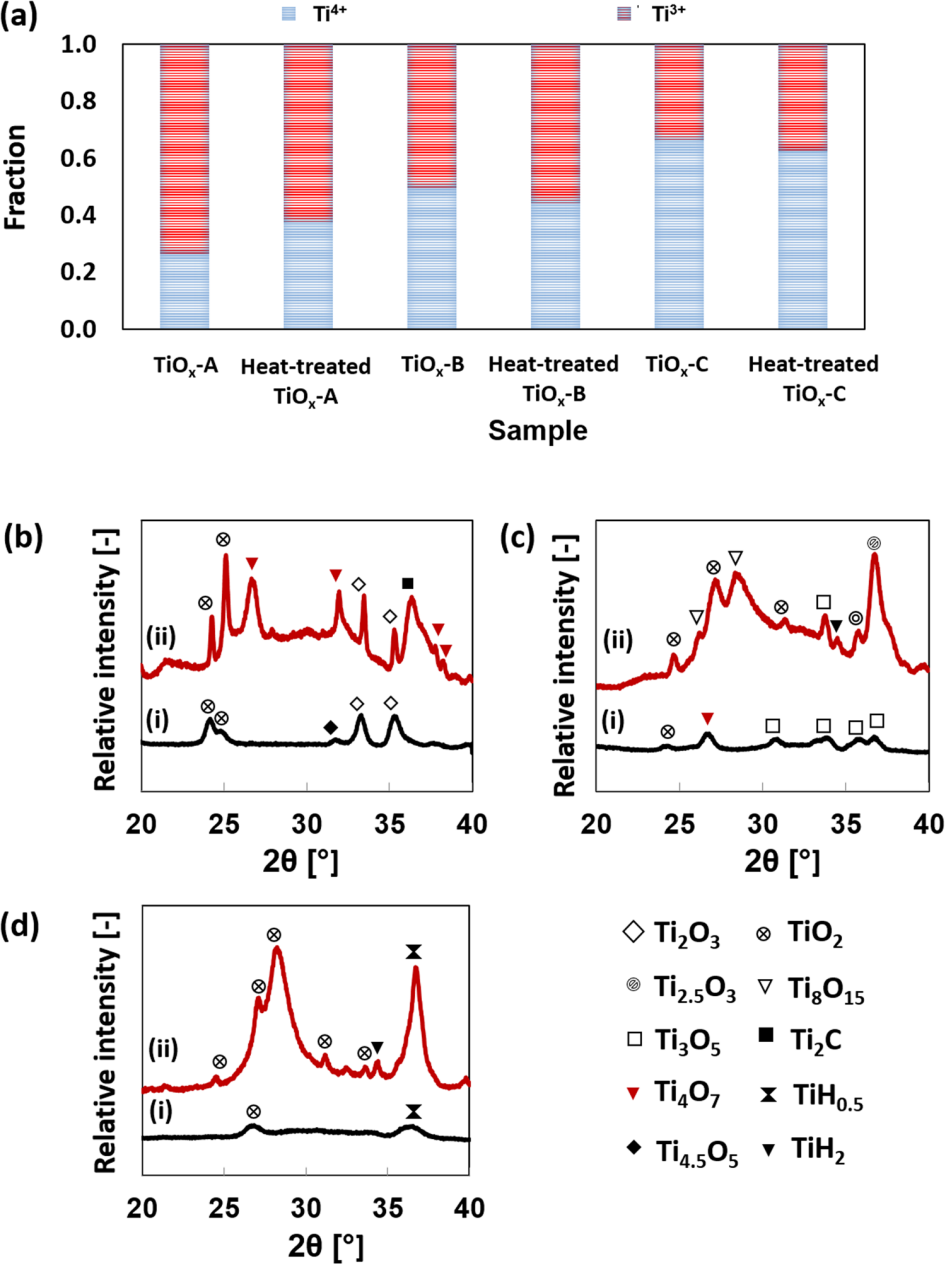
(**a**) Fraction of Ti^3+^ and Ti^4+^ in the samples based on XAFS analysis. The Magnéli phase composition of each sample was determined based on the XRD spectra of (**b**) TiO_x_-A, (**c**) TiO_x_-B, and (**d**) TiO_x_-C. (i) is the spectra of as-synthesized nanoparticles and (ii) is the spectra of heat-treated nanoparticles.

XAFS analysis was performed to confirm the presence of the Magnéli phase. Fitting the XAFS spectra of as-synthesized and heat-treated samples resulted in the fractions of Ti^3+^ and Ti^4+^ before and after heat treatment, as shown in Fig. 5(a). The Ti^4+^ fraction in TiO_x_-B and TiO_x_-C decreased from 49.5% to 44.3% and 66.1% to 62.2%, respectively. In contrast, the Ti^4+^ fraction in TiO_x_-A increased from 26.5% to 37.3% after heat-treatment. Although Ti^3+^ and Ti^4+^ may be associated with Ti_2_O_3_ and TiO_2_, respectively, each fraction may not represent the actual concentration of Ti_2_O_3_ or TiO_2_ because some of the Ti^3+^ and Ti^4+^ may partially form the Magnéli phase. It is clear that, among all of the samples studied in this work, TiO_x_-A has the lowest total Ti^4+^ fraction which decreases the tendency to form TiO_2_ phase.

Interestingly, the Ti^4+^ fraction in TiO_x_-A increased after the heat treatment even though it was performed in a non-oxidizing environment. It is predicted that oxygen from the scission of the –OOC functional group on the particles surface was the source of this partial oxidation. The low-wavelength FT-IR spectrum of TiO_x_-A confirms, after the heat treatment, a significant decrease in the Ti-O-OC broad peak at 1103 cm^−1^ (Figure S2e of the supporting information). Elemental mapping of TiO_x_-A before and after heat treatment also confirms a decreased intensity of carbon element on the particles surface (Figure S2a–d of the supporting information). In an RF induced thermal plasma process, formation of carbides and other carbon functional groups is highly possible when IPA is present in the precursor. The formation of these groups can be suppressed by the addition of water. Because the precursor of TiO_x_-A had the lowest water content, we may expect that TiO_x_-A was enriched with carbon functional groups on the surface. However, further studies are required to confirm this hypothesis.

Aligned with the results from the TEM analysis, the XRD spectra given in Fig. 5(b) to (d) show better crystallinity after heat treatment which eases the identification of the phases. Summary of the phases before and after heat-treatment is provided in Tables S1 to S3 of supporting information. The phases that already exist in the as-synthesized TiO_x_-A were preserved during heat-treatment with a notable increase in Ti_4_O_7_ peak intensity at 26.5° and 32°. On the contrary, a strong peak of Ti_4_O_7_ at 26.5° in TiO_x_-B disappeared after heat-treatment. Some new phases such as Ti_8_O_15_ and TiH_2_ were developed instead. The formation of TiH_2_ in TiO_x_-B is strongly related to the presence of Ti in the as-synthesized particles, which furthermore reacted with H_2_ in the reducing gas. In the heat-treated TiO_x_-C, the broad peak between 27° and 36° almost vanished. At the same time, strong TiO_2_ peaks appeared.

Formation of phases with average intermediate Ti oxidation states (between 3+ and 4+), especially in heat-treated TiO_x_-A, may be correlated to the concept of electrons being shared in the Magnéli phase. The average electronic charge of a Magnéli phase depends on the proportion of Ti^3+^ and Ti^4+^ that form the shear structure by sharing electrons. For example, Ti_4_O_7_ (Ti^3.5+^) consists of two equally populated Ti^3+^ and Ti^4+^, where the electrons in the *d* band are fully delocalized^19^. The formation of a Magnéli phase therefore suggests a rearrangement of Ti atoms due to the heat treatment. This rearrangement may have involved electron transfer between the *d* bands of the Ti atom and partial delocalization of some *d* electrons. The results show a tendency to preserve and/or to form intermediate phases, especially Ti_4_O_7_, when both TiO_2_ and Ti_2_O_3_ were present in the as-synthesized particles. The initiator for the electron excitations is predicted to be the photon energy from heat radiation. Delocalization of some electrons would, furthermore, affect the electron conductivity.

Table 1 compares the electrical resistivity of the sample before and after heat-treatment, measured using the 4-probe method. The resistivity values of the as-synthesized samples are very high, although some conductive Magnéli phases are identified from XRD. One of the reasons for the high resistivity of the as-synthesized powder may be the random lattice structure and the presence of the amorphous phase. It is speculated that the discontinuities in the lattice structure may have inhibited the electron mobility. According to the empirical formula developed by Goodenough, the critical Ti to Ti interatomic distance that determines whether the *d* electrons are collective or localized is 3.01 Å^20^. Above this value, the *d* electrons are localized, indicating a semiconductor state. Therefore, the presence of some lattices with spaces of about 4 Å, as observed in Fig. 2, may indicate that the metal-like phase is interrupted with the presence of semiconducting phases. Due to this phase discontinuity, the resistivity values of the as-synthesized samples were close to the resistivity of known semiconductors.

**Table 1:** Electrical resistivity of the samples before and after heat-treatment.

Sample	Electrical resistivity (Ω.cm)	
	As-synthesized	Heat-treated
TiO_x_-A	1.48 × 10^4^ ± 150	0.04 ± 0.008
TiO_x_-B	3.91 × 10^6^ ± 26458	0.21 ± 0.043
TiO_x_-C	3.65 × 10^5^ ± 1528	0.75 ± 0.125

The electrical resistivity of the samples significantly decreased after heat treatment. TiO_x_-A has the highest electrical conductivity.

Another possible explanation for the high resistivity of the as-synthesized powder is the presence of insulating surface functional groups that increase the contact resistance between particles. An ideal structure of Ti-O on the surface is commonly difficult to realize because the surface of titanium oxide is easily covered by hydroxyl groups that combine with titanium ions^21^. The source of the OH group is likely the water vapour in the air, which made a contact with the particles during the particles evacuation from the particle collector post-synthesis. Plasma-synthesized particles often contain neither oxides nor hydroxides, provided they are confined in the reactor^22^.

The resistivity values after the heat-treatment significantly decreased. This is believed to be the result of several factors. The first is that the presence of Magnéli phases with Ti valences between +3.3 and +3.7. Formation of Magnéli phase enabled partial to complete delocalization of *d* electrons. The presence of Ti_4_O_7_, in which *d* electrons are fully delocalized, is believed to contribute to the high electrical conductivity. As summarized in Table S1 of the supporting information, TiO_x_-A and TiO_x_-C preserved their Ti_4_O_7_ phase with increasing Ti_4_O_7_ peak intensity for TiO_x_-A. It is therefore understandable that heat-treated TiO_x_-A had the lowest resistivity among the heat-treated samples. Meanwhile, the increasing semiconducting phases, *i*.*e*. TiO_2_ and Ti_2_O_3_, resulted in the high resistivity of heat-treated TiO_x_-C compared to the other heat-treated samples, although Ti_4_O_7_ was still present in the heat-treated TiO_x_-C. In contrast with the other two samples, TiO_x_-B lost its Ti_4_O_7_ phase after heat-treatment. Kleberite which is insulating was still present after heat-treatment. However, slightly more resistive phases such as Ti_3_O_5_ and Ti_8_O_15_ which were still present helped improving the conductivity. Titanium hydride formed after heat-treatment of TiO_x_-B and TiO_x_-C were also expected to lower the electrical resistivity^23^.

In addition to the presence of a conducting crystal structure, the significant reduction in the electrical resistivity of the heat-treated samples is likely the result of a lower contact resistance between particles. As confirmed by the FT-IR analysis in Figure S2e, the intensity at around 900 cm^−1^ in the spectrum from insulating aromatic hydrocarbons^24^ of heat-treated samples was lower than that in the as-synthesized sample.

**Figure 6:**
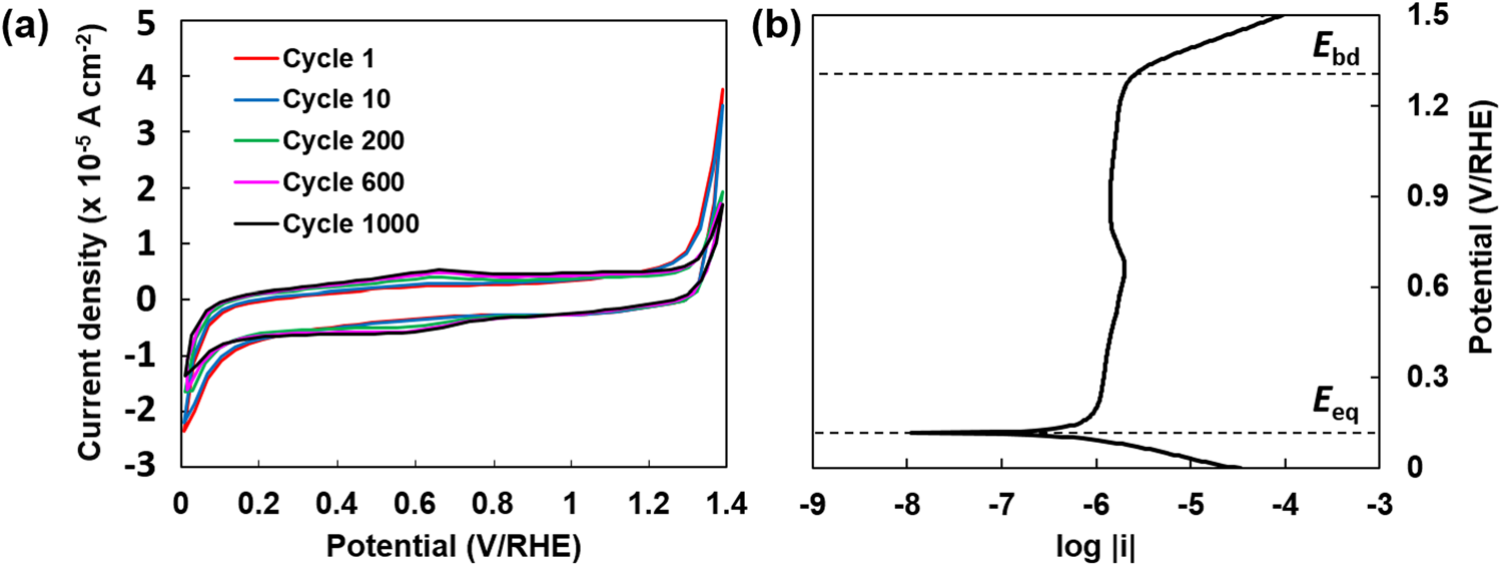
(**a**) Cyclic voltammogram of heat-treated TiO_x_-A in oxygen-saturated HCl 1 M solution between 0 and 1.4 V/RHE at a potential sweep of 50 mV/s. The voltammogram shape did not significantly change after 1000 cycles, especially in the pseudocapacitive region. (**b**) Potentiodynamic curve of heat-treated TiO_x_-A in oxygen-saturated HCl 1 M solution between 0 and 1.5 V/RHE. The curve shows a tendency of TiO_x_-A to passivate with a high break-down potential.

Figure 6(a) shows multiple-cycle cyclic voltammogram of heat-treated TiO_x_-A as the representative sample. The cyclic voltammograms show no visible cathodic nor anodic peak within 0 and 1.4 V/RHE which implies that the heat-treated TiO_x_-A was relatively inert in a strongly oxidizing environment. The overall shape of the voltammogram was preserved after 1000 cycles, indicating the stability of heat-treated TiO_x_-A in a strongly oxidizing environment. Compared to the cyclic voltammogram of Ti_4_O_7_ particles given in Figure S4, the current densities exhibited by TiO_x_-A were lower, likely because of the lower electrical conductivity as a consequence of containing several Magnéli phases. However, TiO_x_-A possesses much better stability over potential cycling than Ti_4_O_7_. Like that found in the voltammogram of Ti_4_O_7_, anodic and cathodic peak at 0.56 V/RHE and 0.66 V/RHE, respectively, appeared after 200 cycles. The precise assignment of these peaks will be a subject for further studies. However, it is predicted that these peaks correspond to the electrochemical reduction of TiO_2_ phase which possibly happens between 0.52 to 0.84 V/standard hydrogen electrodes (SHE)^25^. It is also worth noting that the voltammogram shape is close to square, indicating a double layer charge storage mechanism. Through further functionalization, it is therefore possible to use these nanoparticles as energy storage materials.

The electrochemical stability of heat-treated TiO_x_-A is confirmed by the potentiodynamic curve shown in Fig. 6(b). The equilibrium potential, *E*_eq_, was 0.11 V/RHE, indicated by a substantial decrease of the current density as a response to a small potential elevation. The low *E*_eq_ would imply that the heat-treated TiO_x_-A is easily oxidized under oxygen-rich environment. This value is also lower than that exhibited by Ti_4_O_7_, which was 0.31 V/RHE (see Table S4 for a complete comparison with Ti_4_O_7_). However, Tafel extrapolation on the anodic curve at 60 mV above *E*_eq_ shows a very low exchange current density, 5.37 × 10^−7^ A cm^−2^, which corresponds to a thinning rate of 0.006 mm per year in a continuous exposure to a strongly oxidizing environment. This value is very low that TiO_x_-A can be considered as corrosion-resistant material. The corrosion resistivity is attributed to the passivity of heat-treated TiO_x_-A, indicated by a relatively stable current density above 0.2 V/RHE. The break down potential, *E*_bd_, of the passivity was approximately 1.3 V/RHE, slightly lower than 1.4 V/RHE. The same *E*_bd_ value was also exhibited by Ti_4_O_7_. However, since the cyclic voltammograms of TiO_x_-A show a stable performance within 0 to 1.4 V/RHE up to 1000 cycles, heat-treated TiO_x_ must have had the ability to re-passivate during the backward potential sweep.

## Conclusions

In summary, we have demonstrated the synthesis of Magnéli phases titanium oxide NPs *via* an RF induction thermal plasma method. Nanoparticles with low electrical resistivity were successfully obtained after applying a post-synthesis heat treatment to the as-synthesized NPs. In particular, we found an interesting phenomenon in the formation of new crystal structures after the heat treatment and suggested mechanisms for the crystal structure changes based on the evidence from XAFS and XRD analyses. The resistivity values of the heat-treated samples were related with the Magnéli phase content, especially Ti_4_O_7_, estimated from the XRD spectra. Further investigation of this phenomenon is encouraged for a better understanding of the solid-state transformation of Magnéli phase titanium oxide. The electrochemical characterization shows a remarkable stability of the heat-treated TiO_x_-A NPs in a strongly oxidizing environment, which opens a wider opportunity for the application of plasma-synthesized TiO_x_ NPs.

## Electronic supplementary material

Supplementary Information
