# Supplementary material for: Highly conductive nano-sized Magnéli phases titanium oxide (TiOx)
Source: Sci Rep. 2017 Jun 16;7:3646. doi: 10.1038/s41598-017-03509-y (PMC5473814; doi:10.1038/s41598-017-03509-y)
Supplement: Supplementary file 1 — Supplementary Information [file 41598_2017_3509_MOESM1_ESM.pdf]

Supporting Information

Highly conductive nano-sized Magnèli phases titanium oxide (TiO_x)

Aditya F. Arif[1], *Ratna Balgis*[1], *Takashi Ogi*[1,*], *Ferry Iskandar*[2], *Akihiro Kinoshita*[3], *Keitaro Nakamura*[3], *Kikuo Okuyama*[1]

[1]Department of Chemical Engineering, Graduate School of Engineering, Hiroshima University, 1-4-1 Kagamiyama, Higashi-Hiroshima 739-8527, Japan

[2]Department of Physics, Institut Teknologi Bandung, Jalan Ganesha No. 10 Bandung 40132, West Java, Indonesia

[3]Research Center for Production and Technology, Nisshin Seifun Group Inc., 5-3-1, Tsurugaoka, Fujimino City, Saitama 356-8511, Japan

1. Raw data of XAFS spectra

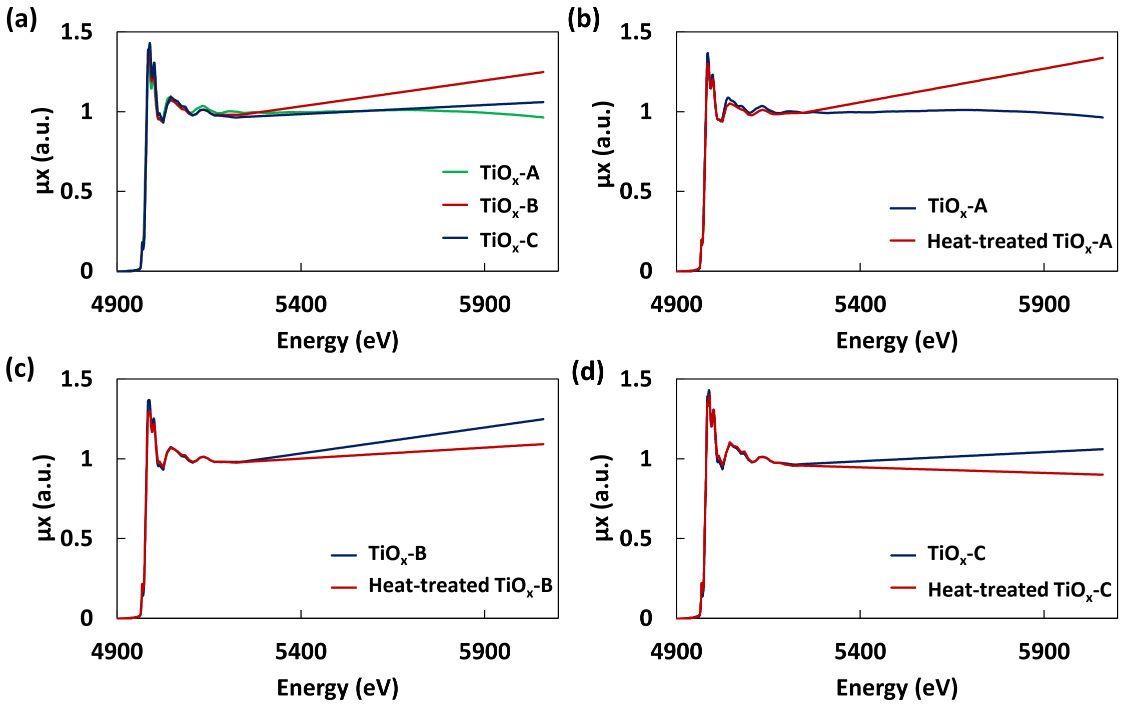

Figure S1. XAFS spectra of (a) TiO_x-A, TiO_x-B, and TiO_x-C, and comparison of XAFS spectra before and after heat-treatment of (b) TiO_x-A, (c) TiO_x-B, and (d) TiO_x-C.

2. Raw data of XAFS spectra

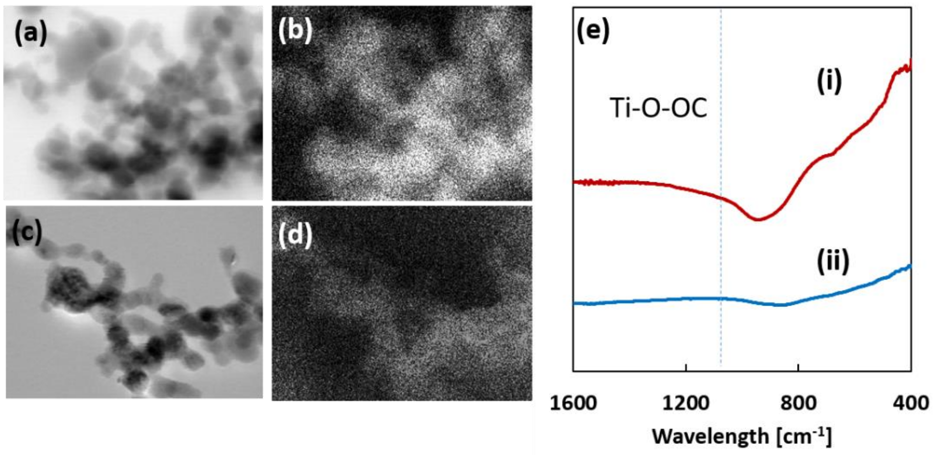

Figure S2. TEM image and carbon elemental mapping of (a, b) TiO_x-A and (c, d) heat-treated TiO_x-A show a reduction in the carbon intensity after heat treatment. This result is aligned with (e) FT-IR spectrum of (i) TiO_x-A and (ii) heat-treated TiO_x-A which show a significant decrease in the Ti-O-OC peak intensity at 1103 cm^{-1} after heat treatment.

3. Phase identification from XRD spectra

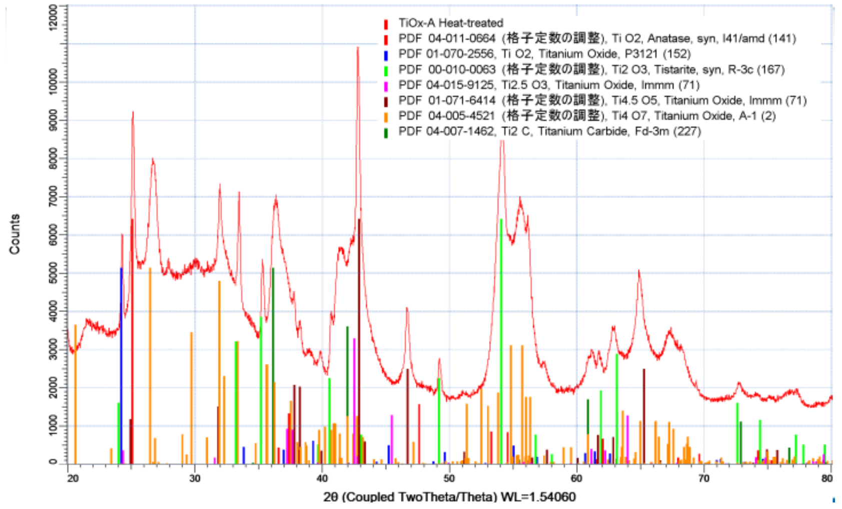

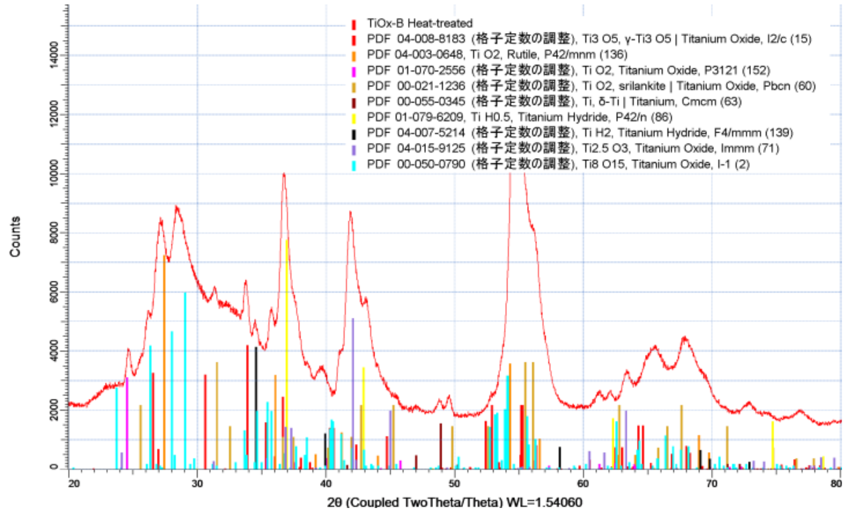

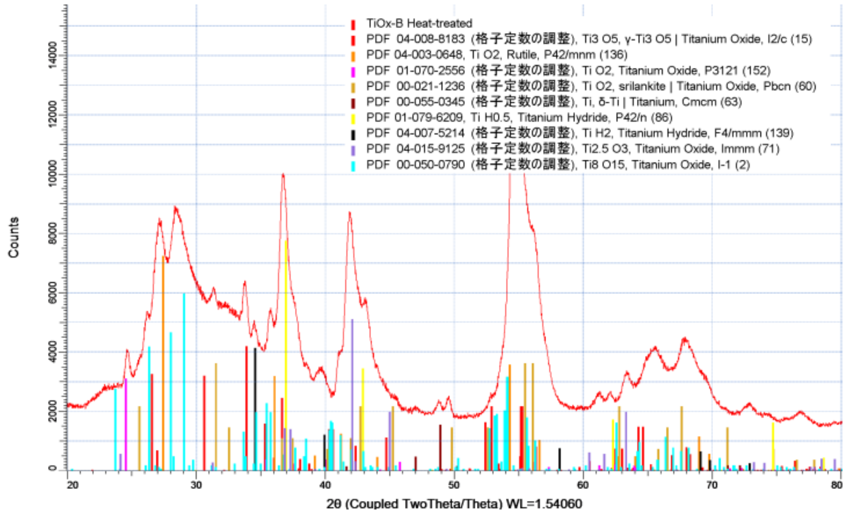

Figure S3. Raw XRD spectra and peak assignment of heat-treated (a) TiO_x-A, (b) TiO_x-B and (c) TiO_x-C

Table S1. Summary of identified phases in TiO_x-A from XRD analysis

Phase	PDF No.	As-synthesized	Heat-treated
TiO_2	04-011-0664	√	√
TiO_2	01-070-2556	√	√
Ti_2O_3	00-010-0063	√	√
$Ti_{2.5}O_3$	04-015-9125	-	√
$Ti_{4.5}O_5$	01-071-6414	√	√
Ti_4O_7	04-005-4521	√	√
Ti_2C	04-007-1462	√	√

Table S2. Summary of identified phases in TiO_x-B from XRD analysis

Phase	PDF No.	As-synthesized	Heat-treated
Ti_3O_5	04-008-8183	√	√
$Ti_{2.5}O_3$	04-015-9125	-	√
TiO_2 (rutile)	04-003-0648	√	√
TiO_2 (P3121)	01-070-2556	√	√
TiO_2 (srilankite)	00-021-1236	√	√
Ti	00-055-0345	√	√
Ti_4O_7	04-005-4521	√	-
$TiH_{0.5}$	01-079-6209	√	√
TiH_2	04-007-5214	-	√
Ti_8O_{15}	00-050-0790	-	√
$Ti_{0.374}O_{0.5}(OH)_{0.5}$ (kleberite)	01-076-5388	√	√

Table S3. Summary of identified phases in TiO_x-C from XRD analysis

Phase	PDF No.	As-synthesized	Heat-treated
Ti_3O_5	04-008-8183	√	√
$Ti_{4.5}O_5$	01-071-6414	√	√
TiO_2 (rutile)	04-003-0648	√	√
TiO_2 (P3121)	01-070-2556	√	√
TiO_2 (srilankite)	00-021-1236	√	√
Ti_2O_3	00-010-0063	√	√

Ti_4O_7	04-005-4521	√	√
$TiH_{0.5}$	01-079-6209	√	√
TiH_2	04-007-5214	-	√
Ti_8O_{15}	00-050-0790	√	-
TiC_8	00-051-0622	-	√
$Ti_{0.374}O_{0.5}(OH)_{0.5}$ (kleberite)	01-076-5388	√	√

4. Electrochemical characteristics of Ti_4O_7 synthesized *via* high temperature H_2 reduction of rutile TiO_2

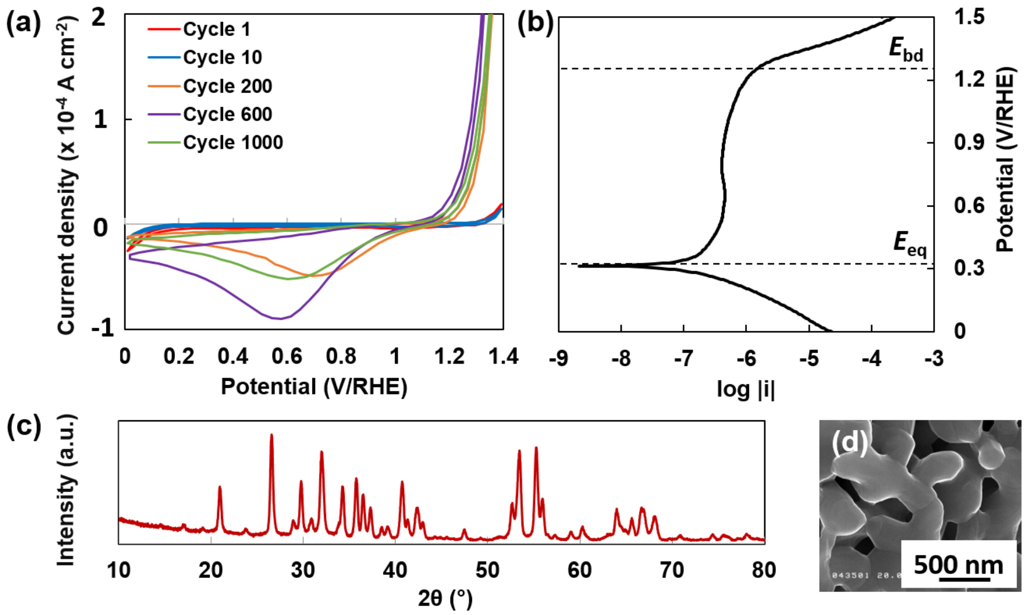

Figure S4. (a) cyclic voltammogram and (b) potentiodynamic curve of Ti_4O_7 in oxygen-saturated 1M HCl. (c) XRD spectra of Ti_4O_7 sample showing Ti_4O_7 peaks (Ref. ICDD No. 50-787) and (d) SEM images of Ti_4O_7 particles

Table S4. Comparison of electrochemical characteristics of TiO_x-A and Ti_4O_7

No	Parameter	Unit	TiO_x-A	Ti_4O_7
1.	E_{eq}	(V/RHE)	0.11	0.31
2.	E_{bd}	(V/RHE)	1.3	1.3
3.	Corrosion current density	A cm^{-2}	5.37×10^{-7}	1.58×10^{-7}
4.	Corrosion rate	mm per year	0.006	0.00005
